# B Lymphocytes: Development, Tolerance, and Their Role in Autoimmunity—Focus on Systemic Lupus Erythematosus

**DOI:** 10.1155/2013/827254

**Published:** 2013-09-26

**Authors:** Gabriel J. Tobón, Jorge H. Izquierdo, Carlos A. Cañas

**Affiliations:** Department of Internal Medicine, Division of Rheumatology, Fundación Valle del Lili, ICESI University School of Medicine, Cra 98 No. 18-49, Cali, Colombia

## Abstract

B lymphocytes are the effectors of humoral immunity, providing defense against pathogens through different functions including antibody production. B cells constitute approximately 15% of peripheral blood leukocytes and arise from hemopoietic stem cells in the bone marrow. It is here that their antigen receptors (surface immunoglobulin) are assembled. In the context of autoimmune diseases defined by B and/or T cell autoreactive that upon activation lead to chronic tissue inflammation and often irreversible structural and functional damage, B lymphocytes play an essential role by not only producing autoantibodies but also functioning as antigen-presenting cells (APC) and as a source of cytokines. In this paper, we describe B lymphocyte functions in autoimmunity and autoimmune diseases with a special focus on their abnormalities in systemic lupus erythematosus.

## 1. Introduction

Systemic lupus erythematosus (SLE) is the prototype of the systemic autoimmune diseases characterized by multiorgan involvement. This systemic compromise is mediated by a global loss of self-tolerance. The loss of tolerance is a consequence of genetic factors, in the context of specific environmental triggers, with the subsequent development of an altered immune response. Both innate and acquired immune mechanisms are implicated in the disease pathogenesis. Recently, special attention has been focused on the B cell abnormalities. In this paper, we will describe the B cell development, tolerance mechanism, and their implications in autoimmune diseases, with emphasis on SLE. 

## 2. B Cell Development and the B Cell Receptor Formation

Different populations of B cells result in preimmune pools where each cell in these quiescent populations expresses a B cell antigen receptor (BCR) with a unique specificity. When the BCRs come in contact with their specific antigen, several intracellular signals are generated leading to activation, differentiation, and formation of plasma cells and memory B cells. This last subset of B cells maintains protective antibody levels and mediates the response to subsequent antigen challenges. As the mechanisms leading to maturing and antibody production are complex, the alterations of some of these populations or critical steps have been associated with immunodeficiency and autoimmune diseases.  Table 1 summarizes the most important features of each of the subpopulations (lineages) of B lymphocytes [1]. 

### 2.1. B Cell Development

This process begins from stem cells present in the bone marrow (BM) which, depending on the different stimuli received, will generate B lymphocytes. They are derived from the early lymphoid progenitor, which passes to the common lymphoid progenitor. This produces, first of all, the natural killer (NK) cells and dendritic cells and, secondly, the common lymphoid-2 progenitor (LCA-2) that is responsible for the B cell lineage, which is considered the first stage of immature B lymphocytes. Development of the B cell lineage depends on BM stromal cells that produce mainly interleukin (IL)-7 but also the Fms-like tyrosine kinase 3 (Flt3-L) and on the action of several transcription factors such as PU.1, IKAROS (IKAROS family zinc finger 1), E2A, EBF (early B cell factor 1), PAX5 (paired box gene 5), and IRF8 (interferon regulatory factor 8) [2–5]. In the BM, B cells pass through several distinct developmental stages. During this, they acquire their antigen specificity, follow a program of differential surface antigen expression and sequential heavy and light chain gene rearrangement, forming the BCR (initially IgM), that determines the cell maturation stage. Reaching the immature stage, B cells exit the BM and complete their development to the mature or naïve stage, which is signaled by the appearance of IgD in addition to IgM on the cell surface. This development sequence occurs in the absence of any contact with exogenous antigen, a stage known as antigen-independent B cell development [2–5]. 

### 2.2. B Cell Receptor Development

**Figure 1:**
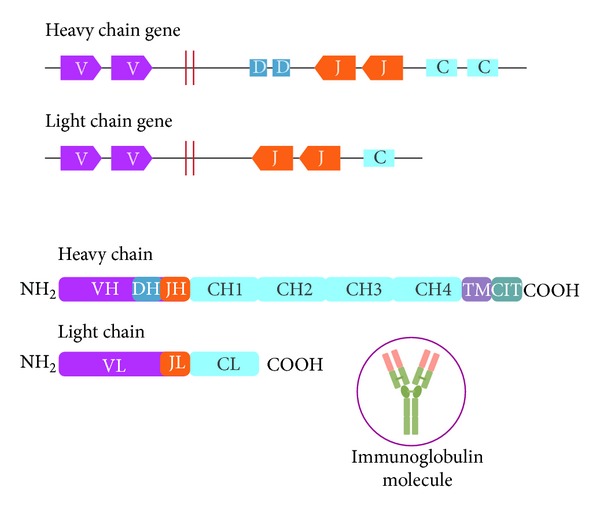
Schematic representation of the components of the H and L chains of immunoglobulins. The light and heavy chain loci are each made up of a series of V (variable) gene elements, followed by several D (diversity) segments (for the heavy chain gene only), some J (joining) segments, and C (constant region) exons. Heavy chains (H) are assembled from 4 segments (VH, D, JH, and CH); light chains (L) are assembled from 3 segments (VL, JL, and CL). The development of the BCR begins when the recombinase enzyme complex catalyzes the fusion of one DH region gene to a JH region gene with the deletion of the intermediate DNA sequences. Next, the recombinase joins one VH region gene to the rearranged DHJH gene. The enzyme terminal deoxynucleotidyl transferase (TdT) is expressed, adding random nucleotides to the sites of VHDHJH joining and enhancing the diversity of amino acid sequences. The rearranged VHDHJH element forms the most 5′ exon of the H chain gene and is followed downstream by exons encoding the constant (C) region (initially *μ* chain), that pairs with an L chain and produces IgM. When the VHDHJH element is followed downstream by exons encoding the C region for the *δ* chain, it produces IgD. These events occur as a result of alternative RNA splicing. Finally, if the rearrangement of VH, DH, and JH elements yields an H chain transcript and encodes a functional H chain protein, this heavy chain is synthesized and pairs in with 2 proteins (called *λ*5 and VpreB), which act as a surrogate light chain, and results in the expression of a pre-BCR. Once a functional heavy chain is produced, the cell downregulates the TdT gene and initiates an L chain rearrangement. It begins first with a *κ* element and, if this rearrangement is unsuccessful, continues with a *λ* element. A V*κ* element rearranges to a J*κ* element and produces a light chain, which, if it is functional, pairs with the H chain to make an immunoglobulin protein.

Immunoglobulin molecules are composed of 2 identical 50 kd heavy chains and 2 identical 25 kd light chains [6]. The genes encoding immunoglobulins are assembled from segments in a manner that is entirely analogous to the process of T cell receptor genes. The light and heavy chain loci are each composed of a series of V (variable) gene elements, followed by several D (diversity) segments (for the heavy chain gene only), some J (joining) segments, and C (constant region) exons. Heavy chains (H) are assembled from 4 segments (VH, D, JH, and CH). Light chains (L) are assembled from 3 segments (VL, JL, and CL) (Figure 1). The genes for 9 different heavy chain types (IgM, IgD, IgG1–4, IgA1-2, and IgE) are located on chromosome 14 and those for 2 light chain types (*κ* or *λ*) are on chromosome 2 and 22, respectively. The variable portions (V) of the H and L chains are in juxtaposition, and this creates the antigen-binding portion of the immunoglobulin molecule. These V regions contain 3 highly variable subregions, or hypervariable sequences, which produce the antigen-binding domain of the molecule. The amino-terminal portions of the chains vary in amino acid sequence from one antibody molecule to another. The carboxyl terminal portions are constant in each subclass of antibody. The H chain constant regions form the Fc domain of the molecule and are responsible for most of the effector functions of the immunoglobulin molecule. 

**Figure 2:**
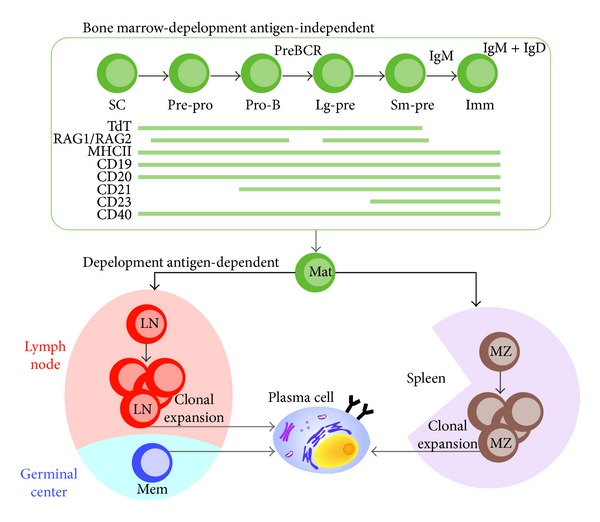
B cell receptor development and differentiation.

The development process of different subsets of B cells has been extensively reviewed elsewhere [4–7] and summarized in Figure 1. Once a functional IgM and IgD are synthesized, the pre-B cell evolves into an immature B cell. The fully mature BCR includes additional transmembrane proteins designated as Ig*α* and Ig*β* that activate intracellular signals after receptor binding to antigen [8, 9]. At that point, the mature B cell passes to peripheral lymphoid tissues (Figure 2).

### 2.3. B Cell Classification according to Their Ontogenic State

As soon as B cells have productively rearranged their immunoglobulin genes, pro-B cells proceed to the pre-B cell stage. On their arrival in the spleen, immature B cells give rise to type-1 (BT1), type-2 (BT2), and possibly type-3 transitional B cells [11]. As transitional B cells, they are pushed into migrating from the BM to secondary lymphoid organs (SLO). Although T1 cells undergo apoptosis in response to BCR engagement, they require signaling via the B cell activating factor belonging to the tumor necrosis factor (TNF) family receptor (BAFF-R, TNFRSF13) to mature to the T2 stage [12]. T2 cells are only present in the spleen and reside in the follicles, whereas T1 cells are found in the red pulp and outer periarterial lymphatic sheath (PALS) [13]. 

**Figure 3:**
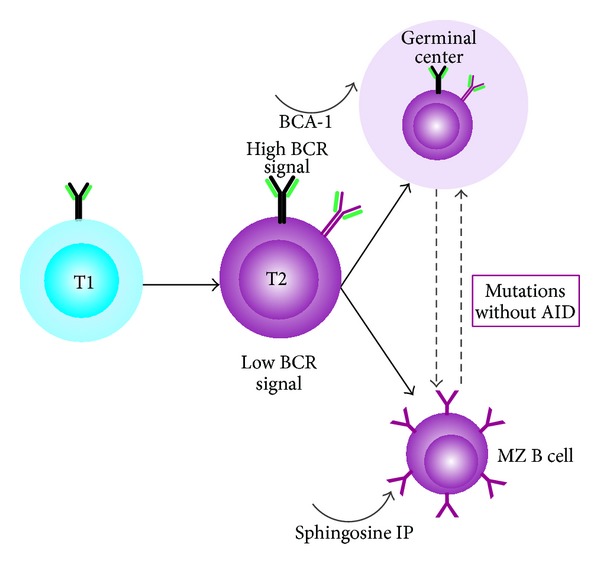
B cell classification based on their ontogenic state. From the transitional type 1 (T1) and T2 B cells, two options depend on the B cell receptor (BCR) evoked signal and the downstream Notch 2 proteins: germinal center (GC) B cells driven by the B cell-attracting (BCA)-1 chemokine (or CXCL13) and MZ B cells with mutations but without activation-induced cytidine deaminase (AID). (Modified from [10]).

There, they continue maturing and are further selected by antigens. As BT1, they present as CD20+CD5+CD10+/−CD21+/−CD23+/−IgM+IgD+/−  and CD38+, but once they have evolved to type 2 (BT2), they become CD20+CD5+/−CD21++CD23+/−IgM++IgD++ and CD38+/−. T2 B cells differentiate into either circulating lymphocytes that get organized as germinal centers (GCs), or noncirculating lymphocytes that populate the marginal zone (MZ). Progression of T2 B cells towards MZ or GCs may be determined by the quality of BCR-evoked signals and the subsequent expression of the Notch proteins [14]. Alternatively, MZ B cells with mutated immunoglobulin genes, but without activation-induced cytidine deaminase (AICDA), may have passed a germinal center (GC) response [15]. Finally, the expression of sphingosine 1-phosphate receptor 1 on the B cells may overcome the recruiting activity of the B cell-attracting chemokine (BCA)-1 to the GCs [16], and thereby retain B cells within the MZ [17] (Figure 3). The main CD molecules expressed by B cells are summarized in Table 2. 

### 2.4. Migration of B Cell into the Germinal Centers

Organization of the B cell follicles and surrounding T cell zones is achieved by the secretion of chemokines by distinct stromal cell subsets. Of these subsets, follicular dendritic cells (FDCs) are essential to retain immune complexes and produce B-lymphocyte chemoattractants (BLC/CXCL13). FDC maintenance requires continual membrane expression of lymphotoxin *α*1*β*2 (LT*α*1*β*2) trimer as well as TNF secretion by B cells and LT*β*R and TNF-R1 expression on FDCs [18]. The MZ demarcates the perimeter of the white pulp of the spleen and contains a subset of B cells that likely arises from the transitional B cell compartment [19]. MZ B cells are strategically located to respond to blood-borne antigens and can rapidly differentiate into antibody-producing cells in the red pulp. Upon an encounter with antigens, follicular B cells migrate to the border regions of the PALS/cortex to present bound peptide and costimulate T cells. Reciprocal B cell activation is mediated by engagement of CD40 and provision of cytokine support. CD40-dependent B cell activation is required to undergo proliferative expansion and differentiation in the GC, where somatic hypermutation and enhanced immunoglobulin class switch recombination (CSR) occur. The architecture of the GC is divided into distinct regions: rapidly dividing B cells or centroblasts in the “dark zone” of the GC give rise to centrocytes which occupy the “light zone.” The light zone is thought to be the site of B cell selection by FDC-bound antigens that are processed and presented by B cells to primed T cells of the follicular helper CD4+ (Tfh) subtype. 

B cell maturation in the GC is accompanied by somatic hypermutation of antibody variable region (V) genes, which provides the molecular basis for the production of B cells bearing high-affinity antigen receptors. These B cells are thought to have a competitive advantage when antigen becomes limiting and GC structures present atrophy. B cells unable to bind antigen or receive sufficient T cell help die *in situ* by apoptosis and are cleared by macrophages, whereas antigen-selected B cells that leave the GC become memory B cells or plasmablasts by a process that is not fully understood. Long-lived plasma cells are actively retained in the BM responding to stromal derived factor/CXCL12 as well as survival factors such as IL-6, B cell activating factor (BAFF), and a proliferation-inducing ligand (APRIL). The trafficking of B cells in the lymphoid organs and target tissues is a regulation mechanism of B cell activation and differentiation [20–22].

**Figure 4:**
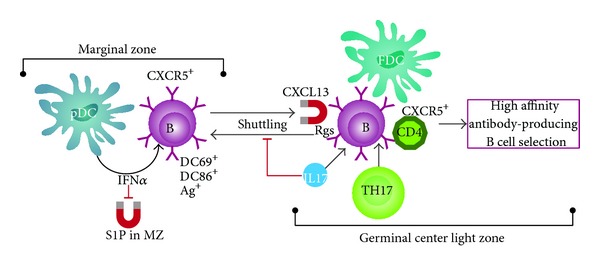
Chemotactic responses play a key role in orchestrating the cell-cell interactions in the germinal centers.

B cells can act as an antigen delivery system that transports blood-borne antigens into the FDC network region of the spleen [17]. This regulates the GC formation where high affinity antibody-forming B cell differentiation occurs. These migratory responses are extremely dynamic and involve ongoing shuttling of the B cells between the different anatomic sites and the GCs. Chemotactic responses play a key role in orchestrating the cell-cell interactions in the GCs. This process involves ongoing shuttling of the antigen-carrying B cells between the MZ and the GCs. In animal models of autoimmunity, the migration of MZ precursor B cells is promoted by high levels of interferon (IFN)-*α* produced by plasmacytoid dendritic cells (pDC) in the marginal sinus that antagonize the activity of the S1P1 chemokine receptor. In contrast, within the GCs, IL-17A upregulates the expression of regulators of G protein signaling (RGS) in B cells to desensitize the G protein-coupled receptor (GPCR) signaling pathway of CXCL12 and CXCL13 chemokines [23–25]. This provides a prolonged stable interaction of B and T cells in the GC that induces high levels of AICDA and, as a result, enables the development of pathogenic autoantibody-producing B cells (Figure 4).

### 2.5. Mature B Cells

Peripheral B cell maturation, homeostasis, and antigen-dependent differentiation are complex processes occurring in distinct anatomic locations. As B cells egress from the BM, further maturation into follicular or MZ B cells is dependent upon the effects of the cytokine BAFF. B cell compartmentalization and cell-cell interactions in the SLO require expression of membrane-bound LT*α*/*β* trimers and TNF, whereas T cell-dependent B cell differentiation requires engagement of CD40 (TNFRSF5) by CD40L on activated CD4+ T cells. CD30 (TNFRSF8) is expressed on activated B cells and has been found to be required for efficient memory B cell generation. CD27 is also implicated in B cell memory.

**Figure 5:**
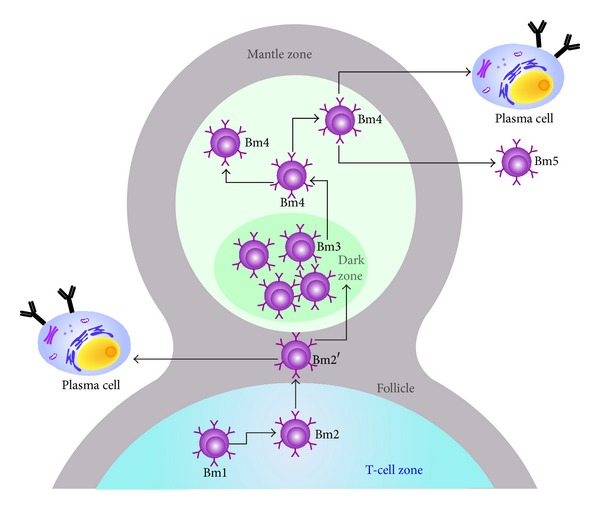
Germinal center (GC) changing a primary lymphoid follicle (LF) into a secondary LF. The GC is surrounded by the mantle zone, which is comprised of the light and dark zone, and populated by mature B (Bm) cells evolving from Bm1 in the T cell area through plasma cells that come back to the bone marrow.

The development stages of GC B cells are based on the relative expression of IgD and CD38 on mature B (Bm) lymphocytes [26] from naïve cells leaving the BM (Bm1) to memory B cells activated and differentiated by their specific antigen (Bm5). The development starts with CD38−IgD+ naïve Bm1 that progresses into CD38+IgD+ antigen activated Bm2, of which some become CD38++IgD+Bm2′ GC founder cells. These differentiate into CD38++IgD−Bm3 centroblasts and Bm4 centrocytes (Figure 5). Two types of B cells arise from GC reactions: CD38+IgD− early memory B cells that mature locally into CD38−IgD−Bm5 memory B cells and CD38++IgD− plasmablasts, which were first described by Odendahl et al. [27]. The latter return to the BM where they differentiate into long-lived plasma cells. A few cells of each subset escape into the circulation from GCs. 

### 2.6. B Cell Distribution Abnormalities in Systemic Lupus Erythematosus

Several studies show differences of certain peripheral B cell subsets in SLE patients compared to healthy controls. Populations such as transitional B cells (CD24++CD38++), prenaïve and naïve B cells are expanded in the peripheral blood of patients [28], indicating a population shift within the preimmune B cell compartment toward the more immature B cells. Whether these abnormalities reflect an intrinsic B cell defect or are secondary to inflammation or immune deregulation is unclear, but the excess of some cytokines such as BAFF may explain part of these differences. In peripheral blood of healthy controls transitional B cells account for only 2 to 3% of all B cells [29, 30]. In contrast, SLE patients have an increased frequency of approximately 6-7%. This high proportion does not correlate with disease activity and titres of autoantibodies. Due to the lymphopenia seen in SLE patients, the absolute number of transitional B cells is not different to that of controls. The most important check point in SLE seems to be at the transitional stage. High number of self-reactive mature naïve B cells which subsequently originate autoantibody producing plasma cells. This is the most reported characteristic of the abnormal B cell homeostasis in SLE characterized by the expansion of peripheral CD27++ plasmablasts [31], which also correlates with disease activity and the titre of autoantibodies [32]. On the other hand, the frequency of CD19+CD27+ memory B cells seems to be unaffected in SLE patients with active and inactive disease, although the total number of memory B cells is decreased in SLE patients compared to healthy controls [27]. 

### 2.7. B Cell Derived Cytokines

IL-7 is important in B cell functioning. This cytokine plays several important roles during B cell development including aiding in the specification and commitment of cells to the B lineage, the proliferation and survival of B cell progenitors, and maturation during the pro-B to pre-B cell transition [33]. Regulation and modulation of IL-7 receptor (IL-7R) signaling is critical during B lymphopoiesis because excessive or deficient IL-7R signaling leads to abnormal or inhibited B cell development [34]. IL-7 works together with *E2A*, *EBF*, *Pax-5*, and other transcription factors to regulate B cell commitment while it also works to regulate immunoglobulin rearrangement by modulating *FoxO* protein activation and *Rag* enhancer activity. Suppressors of cytokine signaling (SOCS) proteins are inhibitors of cytokine activation and, in B cells, function to fine-tune IL-7R signaling. This ensures that appropriate IL-7 signals are transmitted to allow for efficient B cell commitment and development [35].

Recent discoveries have unveiled new insights into B cell derived cytokines, including IFN-*γ* and IL-4 that modulate the response [36]. They are likely to serve as effectors of some B cell functions. Given the kinetics of B cell generation and the cytokine profile of B lymphocytes, T helper (Th) 1 phenotype may be imprinted by B effector (Be) 1 cells through the expression of IL-2 and IFN-*γ* by B cells. This is sustained by an IFN-*γ*/IFN-*γ* receptor autocrine loop. Conversely, Th2 cells induced naïve B cell polarization into Be2, which produces IL-4 and IL-6 in the absence of GATA-3. In fact, the Th1/Th2 cytokine balance changes with the progress of the immunopathological lesions on autoimmune diseases such as SLE and primary Sjögren's syndrome [37]. Distinct populations of serum cytokines have also been found to differentiate autoimmune disease patients from controls and one patient from another depending on the presence or absence of different organ involvement [38]. B cell produced cytokines may be classified as proinflammatory (IL-1, IL-6, TNF-*α*, and LT-*α*), immunosuppressive cytokines (TGF-*α* and IL-10), or as hematopoietic growth factors (granulocyte/monocytes-colony stimulating factor and IL-17).

### 2.8. B Cell Transcription Factors

B cell development depends on several transcription factors. One of the most important transcription factors is *Pax5*. *Pax5* restricts the developmental potential of lymphoid progenitors to the B cell pathway by repressing B-lineage-inappropriate genes while it simultaneously promotes B cell development by activating B-lymphoid-specific genes. Therefore, *Pax5* controls gene transcription by recruiting chromatin-remodeling, histone modifying, and basal transcription factor complexes to their target genes [39]. Moreover, *Pax5* contributes to the diversity of the antibody repertoire by controlling VH-DJH recombination. It does this by inducing contraction of the immunoglobulin heavy-chain locus in pro-B cells, which is likely mediated by PAIR elements in the 50 region of the VH gene cluster. Importantly all mature B cell types depend on *Pax5* for their differentiation and function. *Pax5* thus controls the identity of B lymphocytes throughout B cell development. Consequently, conditional loss of *Pax5* allows mature B cells from peripheral lymphoid organs to develop into functional T cells in the thymus via differentiation to uncommitted progenitors in the BM. *Pax5* has also been implicated in some diseases including human B cell malignancies.

## 3. B Cell Tolerance Mechanisms and Their Role in Autoimmunity

### 3.1. B cell Tolerance

This mechanism is essential for maintaining nonresponsiveness to thymus-independent self-antigens such as lipids and polysaccharides. B cell tolerance is also important in preventing the development of antibody responses to protein antigens. Both central and peripheral mechanisms are implicated in B cell tolerance. In the central tolerance, the immature B lymphocytes that recognize self-antigens in the BM with high affinity are deleted or activate mechanisms to change their specificity by receptor editing. This fate is defined by the strength of BCR signaling: a strong BCR signal by binding with high affinity to an autoantigen will lead to deletion or receptor editing (see below) while an intermediate binding affinity will permit B cells to survive and continue to the periphery [40].

If a mature B cell recognizes autoantigens in peripheral tissues without specific helper T cell response, this cell may be functionally inactivated by anergy mechanisms or die by apoptosis. The AICDA is required for B cell tolerance in humans. This enzyme is required for CSR and somatic hypermutation. Patients with AICDA deficit develop primary immunodeficiencies and autoimmune complications. Single B cells from AICDA-deficient patients show an abnormal immunoglobulin (Ig) repertoire and high frequencies of autoreactive antibodies [41]. 

### 3.2. B Cell Receptor Editing

When the B cell differentiation is ongoing, its receptor presents a phenomenon known as receptor editing, which is the process of antibody gene rearrangement to have a functional BCR and inhibit further rearrangement (allelic exclusion). The receptor editing is a major mechanism of central tolerance in B cells. If a T lymphocyte produces a self-reactive receptor, different mechanisms are initiated to induce the apoptosis of this self-reactive cell (negative regulation). However, B cells have a second chance at escaping this negative regulation by “editing” the specificities of their receptors with additional antibody gene rearrangements. Immature B cells in the BM that encounter multivalent self-antigens revert to pre-B stage and continue to rearrange *κ* and, if necessary, *λ* light chain genes and generate newly generated B cells that have a novel light chain that is no longer self-reactive. In this case, immature B cells with novel light chains that are no longer part of a self-reactive BCR migrate to the periphery as BT1 cells where they mature into newly generated IgM and IgD expressing recirculating BT2 cells and, then, into mature recirculating B cells. Furthermore, edited B cells are not simply endowed for life with a single, invariant antigen receptor, because an edited B cell whose initial *Ig* gene is not inactivated during the editing process may exhibit two specificities [42].

The BCR editing process initiates with the allelic exclusion. This is the phenomenon in which B cells usually express a single kind of antibody H chain and L chain, and it is typically enforced at the genetic level with only one allele being productively rearranged. A series of epigenetic mechanisms, including replication timing, DNA methylation, histone modification, nucleosome positioning, and heterochromatization, appear to control H and L chain locus accessibility and which allele is first rearranged [43]. These mechanisms regulate accessibility to recombination machinery and activate feedback inhibition of the rearrangement between H chain and L chains. Once the H chain protein is completed, L chain rearrangements initiate. This process is regulated by isotypic exclusion, a phenomenon in which B cells usually express a single L chain isotype (either *κ* or *λ*, not both) and is explained by two properties of L chain rearrangement: first, the *κ* or *λ* rearrange at different times during B cell development, and second, the B cells which express *λ* often have both *κ* alleles deleted. Based on the analysis of cell lines in mouse and human, it was clear that *κ* chain nearly always rearranges before *λ* chain [44, 45].

**Figure 6:**
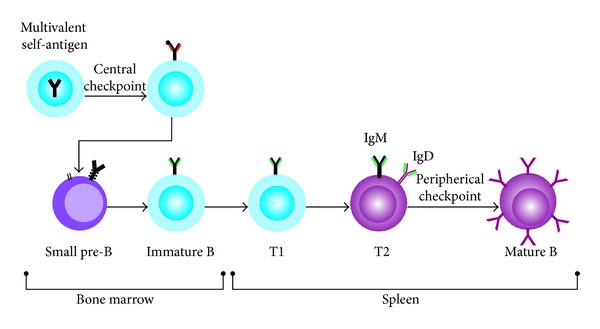
Receptor editing as a major mechanism of central tolerance in B cells. Receptor editing is a major mechanism of central tolerance in B cells. Immature B cells in the bone marrow that encounter multivalent self-antigens revert to the small pre-B stage, continue to rearrange k and, if necessary, l light chain genes, and generate newly B cells that have a novel light chain that is no longer self-reactive. Immature B cells with novel light chains that are no longer part of a self-reactive B cell receptor then migrate to the periphery as T1 B cells where they mature into newly generated IgM and IgD expressing recirculating T2 B cells and, then, into mature recirculating B cells.

Another process identified is the secondary rearrangement of H and L chains. In heavy chain, the mechanism is mediated by DH-JH rearrangement, DH-DH fusion, and VH replacement, all of which contribute to the elongation of the third complementarity determining region (CDR3) and promote autoreactivity. During DH-JH rearrangement, a DH gene upstream of the existing DH-JH rearrangement recombines with a JH gene downstream of the DH-JH rearrangement and replaces it by a leapfrogging deletion rearrangement. In a DH-DH fusion, the recombination process links a 5′ DH segment to a preceding DH-JH rearrangement rather than to a 3′ JH gene. DH-DH fusion occurs more frequently in murine lupus than in nonautoimmune strains of mice [46, 47]. Finally, during VH replacement, the conventional 23 recombination signal sequence (RSS) of an upstream murine VH undergoes RAG-dependent deletional rearrangement with the cryptic RSS of an existing downstream VH gene which is part of an existing VDJ rearrangement on the same allele. This rearrangement results in replacement of all but the very 3′ end of the previously rearranged VH with a new VH. Secondary rearrangement, which would consist of either deletion or inversion of the chromosomal DNA between the recombining gene segments, can also occur at the *κ* locus. These rearrangements are apparently part of an important physiological process underlying failed allelic exclusion and might occur to edit the specificity of a self-reactive BCR (Figure 6).

### 3.3. Control of Receptor Editing

Receptor editing has a genetic control and has been studied in several models. Pre-B cells expressing I*κ*B show evidence of receptor editing which is consistent with a role for *NF*κ*B* [48]. *PLC*γ*2* is present in higher quantities in immature B cells, showing increased phosphorylation in response to BCR crosslinking and probably induces the expression of* Rag2* in these cells. However, other data show downregulation of rag induced by PLC*γ*2 and thus terminate receptor editing. Immature B cells can be induced to edit by BCR crosslinking while transitional B cells cannot. This may be due to an altered signaling pathway through *PLC*γ*2* [49, 50].

The mechanisms that suppress editing and their potential role in autoimmune diseases are under research. 

### 3.4. B Cell and Autoimmunity

Classically, the immune mechanisms implicated in the development of autoimmune diseases have been categorized into two broad sets of diseases: one set in which the pathological process is driven by T cells and the other in which the humoral B response mediates the disorder by producing autoantibodies that are able to bind tissue self-antigens or by forming immune complexes. In recent years, with the new knowledge about the immune response, this approach—dividing autoimmune diseases into T cell and B cell mediated diseases—has dramatically changed. It is now recognized that T lymphocytes facilitate adaptive immune B responses, and B cells play a reciprocal role during CD4 T cell activation in autoimmune diseases. 

For instance, most disease-related autoantibodies are IgGs that are somatically mutated, and this suggests that helper T cells drive the autoimmune B cell response [51]. In addition, B cells have been shown to be important mediators of some autoimmune diseases. These are classically described as T cell mediated and include rheumatoid arthritis (RA), multiple sclerosis (MS), and type 1 diabetes mellitus (T1D). In diseases in which specific autoimmune T cell clones drive the process of inflammation, autoantibody synthesis may represent a marker for the expansion of autoantigen specific B cells that capture and present autoantigen peptides to T cells. As mentioned before, the central tolerance mechanisms are crucial in preventing B cell mediated autoimmune diseases. For instance, the strong BCR signal from binding with high affinity to an autoantigen will lead to deletion or receptor editing of the high affinity. This concept has been demonstrated in several autoimmune animal models, including a double-transgenic mouse model carrying not only the heavy chain against the myelin oligodendrocyte glycoprotein (MOG) autoantigen but also the light chain. The authors demonstrated that B cells expressing solely the MOG-specific Ig H chain differentiate without tolerance. On the other hand, double-transgenic B cells expressing transgenic Ig H and L chains are subjected to receptor editing [52, 53]. 

If the signaling potential of the BCR is affected, for example, by overexpression of CD19 or *Ptpn22* polymorphisms (described in several autoimmune diseases), the self-reactive B cells will not be deleted and may reach the periphery [54, 55]. These mechanisms lead to the increase of self-reactive B cells in the periphery and, as a consequence, the possibility of developing autoimmune diseases. Thus, leaky central tolerance increases the risk for subsequent development of autoimmune disease, but additional factors (genetic, hormonal, environmental, etc.) control this progression from autoimmunity to autoimmune disease.

The role of Toll-like receptors (TLR) in B cell and autoimmunity has also been explored. In a study to determine the stimuli contributing to the development into MZ B cells (involved in autoimmunity), TLR9 stimulation by CpG of transitional B cells induces proliferation and specific maturation into B cells with phenotypic markers of MZ B cells. Also the terminal differentiation into antibody-secreting cell was triggered, leading to autoantibodies synthesis. On the other hand, mature B cells do not differentiate into MZ following TLR9 stimulation. These results suggest that transitional B cells are specifically sensitive to TLR9 stimulation to induce autoreactive B cells [56]. 

### 3.5. B Cell Functions in Autoimmunity

B cells do not simply produce autoantibodies. In fact, B lymphocytes are uniquely endowed to drive autoimmunity as APC because they can bind native self-proteins through their BCR, process them, and present them to T lymphocytes. To demonstrate the antigen-presenting effect of B cells in autoimmunity, several models and observations have been used. For example, in the murine experimental allergic encephalomyelitis (EAE), B lymphocytes are dispensable when disease is induced by MOG peptides but absolutely required for disease to develop if mice are immunized with MOG protein [57]. In MOG-specific TCR and BCR double-transgenic mice, self-reactive B cells cause severe EAE by presenting endogenous MOG protein to self-reactive T cells rather than by autoantibody production [58, 59]. In addition to this observation in EAE (a classical described T cell disease), B cell depletion by rituximab strongly reduced disease severity, affecting the delayed type hypersensitivity and reducing T cell proliferation and IL-17 production [60]. The IL-6 seems important to mediate these effects as indicated by the findings that rituximab effects are not observed in IL-6 KO mice with EAE. 

Another example to show that B cells functions in autoimmunity are not only producing autoantibodies is the transgenic mIgM.MRL-FASlpr mouse. In this model, whose B lymphocytes cannot secrete antibodies but can present antigen, lupus develops spontaneously and T cell activation is comparable to MRL/lpr controls [61]. Likewise, nonobese diabetic (NOD) mice with a mutant IgM heavy chain that cannot be secreted demonstrate that increased insulitis and spontaneous diabetes may occur in the absence of antibody production but require antigen presentation by B cells [62].

The ability of B cells to bind autoantigens through their BCR allows them to act as potent APCs at very low protein concentrations. In the MOG-specific TCR and BCR double-transgenic mice, antigen specific B cells process and present MOG protein to T cells at concentrations that are 100-fold lower than B cells with other BCR specificities. Other functions of B cells are cytokine and chemokine synthesis and ectopic lymphoid neogenesis in autoimmune diseases. 

### 3.6. Amplification of the Autoimmune Response by Epitope Spreading

B cells bind to a specific epitope in antigens via their BCR. After the initial recognition, protein and even protein complexes can be internalized and processed for antigen presentation. The protein may, however, contain several other epitopes besides the epitope originally recognized by the BCR, which can fit in the binding grooves of the MHCII molecules in the B cell. As a consequence, the B cells can present not only the original epitope but also other epitopes of the same protein or protein complex to T lymphocytes and thereby trigger different T cell specificities [63]. This phenomenon, known as epitope spreading, allows autoantigens that were not the initial targets of autoreactive lymphocytes at the onset of autoimmunity to become antigens at later stages [64]. This phenomenon is described in almost all immune diseases and is frequently associated with disease progression [64]. Epitope spreading may trigger the clinically manifested autoimmune disease. As a representative example, the SJL/J mice immunized with protelipid (PLP) proteins develop T cell responses specific to different epitopes in the molecule. These distinct T cell responses contribute to the relapse phases of the EAE and can initiate disease upon secondary adoptive transfer to naïve animals [65]. Epitope spreading also occurs in the NOD mouse model of spontaneous diabetes. In this model, T cell responses and antibodies to type 1 diabetes (T1D), autoantigens, GAD65 and GAD67 isoforms of GAD are observed in mice at 4 weeks of age. At 6 weeks of age, T and B lymphocyte responses for other *β* cell antigens—peripherin, carboxypeptidase H, and Hsp60—are also detected. By 8 weeks of age, responses to all former antigens are enhanced. The initial GAD specific reactivity in this model coincides with the onset of insulitis whereas the progression of insulitis to *β* cell destruction with age correlates to the epitope spreading of B and T cells [66]. Temporal progression of autoreactivity to autoimmune disease by epitope spreading also occurs in human autoimmune diseases. In childhood T1D diabetes, insulin autoantibodies (IAA) are the first autoantibodies detected. IAA-positive children that sequentially develop antibodies to other *β* cell antigens such as GAD and protein tyrosine phosphatase-like proteins IA-2 usually progress to T1D. In contrast, children that remain positive for only IAAs rarely develop the disease [67]. In RA, several reports have shown that the number of antibody specificities increases over time. Like T1D patients, healthy individuals with a broad anticitrullinated peptide antibody (ACPA) profile have a higher risk of developing arthritis [64, 68]. This phenomenon is also observed in SLE patients. In this case, the number of positive antibodies in serums of patients also increases over time until the onset of clinical symptoms as demonstrated in the classic article about autoimmune diseases prediction by Arbuckle et al. [69]. 

### 3.7. The Effects of the Cytokine BAFF in B Cell Tolerance and SLE Development

**Figure 7:**
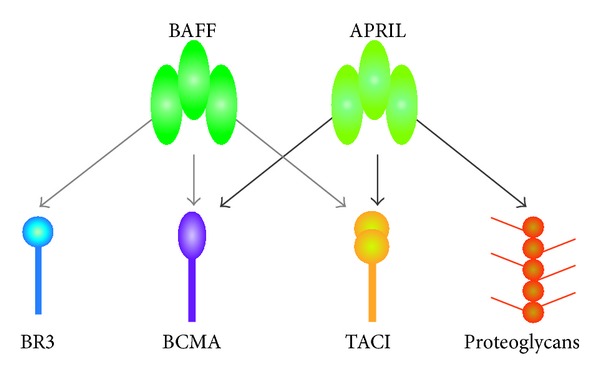
BAFF and APRIL receptors. BAFF binds chiefly to BAFF-R (BR3) but also to BCMA and TACI. APRIL, in turn, interacts with TACI and BCMA, but not with BR3. In addition, APRIL binds to proteoglycans expressed in membranes of lymphoid and nonlymphoid cells.

The cytokine BAFF (for B cell activating factor belonging to the TNF family) has emerged since 1999 [70] as one of the critical factors controlling B cell maturation, tolerance, and malignancy. BAFF plays a key role in B cell differentiation, survival, and activation [70]. BAFF, also known as B lymphocyte stimulator (BLyS), is a cytokine that prevents apoptosis of autoreactive B cells [21]. The BAFF family consists of two ligands, a proliferation-inducing ligand (APRIL) and BAFF; and three membrane receptors, BCMA (B cell maturation antigen), TACI (transmembrane activator, calcium modulator, and cyclophylin ligand interactor), and BAFF-R (also known as BR3). The interactions between ligands and receptors vary: thus, BAFF interacts chiefly with BR3 but can interact with all three receptors, whereas APRIL can interact with TACI and BCMA, but not with BR3 [71]. BAFF enhances B cell survival, drives B cell maturation especially at the early transitional stages, and discontinues humoral tolerance by rescuing autoreactive B cells from apoptosis [72].  Figure 7 shows the different receptors for BAFF and APRIL.

### 3.8. Double-Transgenic Mice Expressing Both HEL and Anti-HEL B Cell Receptor

**Figure 8:**
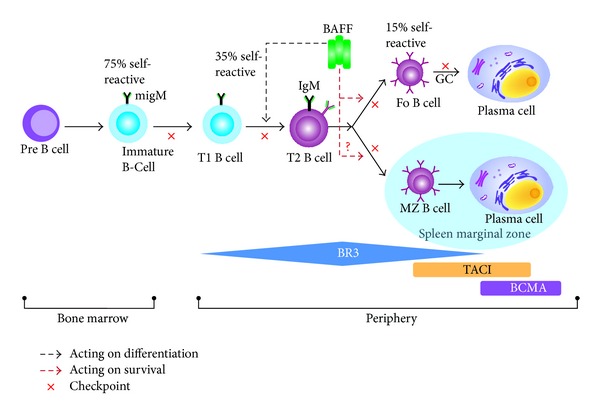
BAFF receptor cell surface expression and self-tolerance during B cell ontogenesis. Data indicate the proportion of self-reactive B cells at specific B cell stages before or after checkpoints as determined in the anti-HEL/HEL transgenic models. Fo: follicular; GC: germinal center; Imm: immature; MZ: marginal zone; Pre: precursor; T1 or 2: transitional type 1 or 2.

As mentioned before, to avoid the generation of pathogenic autoantibodies, self-reactive lymphocytes have to be deleted or anergized at successive immune checkpoints during B cell development and maturation. Because immunoglobulin gene rearrangement is a random mechanism, 50–75% of the newly generated B cells in the BM have a self-reactive BCR. However, the development of autoimmune disease is rare, affecting up to 5% of the population. Consequently, effective mechanisms exist for preventing immune activation of self-reactive lymphocytes. BAFF is known for its role in the survival of mature B cells. Based on its receptor expression profile, BAFF has no effect on B cell tolerance in the BM but does act at the periphery (Figure 8). BAFF certainly plays a major role in B cell tolerance after the BT1 immature B cell stage. Whether or not BAFF can influence self-reactive BT1 cell elimination is unclear. However, BAFF is certainly needed for the survival of BT2 cells and downstream B cell subsets. BT2 cells, which express high levels of BAFF-R, are indeed dependent on BAFF because of their propensity for apoptosis [73], and B cell ontogenesis is stopped at the T1 stage when BAFF or BAFF-R are lacking [74]. One of the most informative systems for studying B cell tolerance is the double-transgenic (Tg) mouse model which expresses the anti-hen-egg lysozyme (HEL) BCR and HEL simultaneously. When HEL is expressed as a cell surface molecule, self-reactive B cells are deleted or undergo additional *ig* gene rearrangements by the receptor editing mechanisms. When HEL is expressed as a soluble protein (sHEL), self-reactive B cells can migrate into the periphery where their fate depends on their ability to compete with non-self-reactive B cells. Without competition, self-reactive BT2 cells persist in an anergic state. In the presence of competition, self-reactive BT2 cells need the cytokine BAFF to sustain their survival and maturation. Because BAFF levels are limited under normal conditions, these self-reactive B cells undergo apoptosis. Thus, if double Tg mice for sHEL/anti-HEL are treated with antagonist for BAFF, survival of sHEL self-reactive B cells is dramatically decreased [75]. In contrast, when BAFF is overexpressed, sHEL self-reactive BT2 cells survive and colonize follicles and MZ in the spleen [76]. Of note, when anti-HEL B cells compete with normal B cells in the animal, excessive expression of BAFF no longer prevents the escape of self-reactive B cells. In this scenario, self-reactive cells are eliminated at a much earlier maturation stage (T1), a stage when B cells express little BAFF-R and as such are unable to sense excessive BAFF production that can only efficiently rescue BT2 cells.

### 3.9. BAFF-Transgenic Mice

BAFF-Tg mice constitute an effective model for autoimmunity. Overproduction of BAFF in these mice leads to B cell proliferation, auto-antibody production, and, ultimately, development of kidney failure similar to SLE-associated symptoms. Moreover, aging BAFF-Tg mice also present a primary Sjögren's syndrome-like disease, in which they demonstrate inflammation and destruction of salivary glands (SGs) [72]. In addition to the attendant polyclonal hypergammaglobulinemia, BAFF-Tg mice develop elevated titers of multiple autoantibodies, including antinuclear antibodies, anti-double-stranded DNA, rheumatoid factors, circulating immune complexes, and immunoglobulin deposits in kidneys. Some B cell subsets such as BT2 cells, follicular (Fo) B cells, and MZ B cells rise. Moreover, without stimulation, a high number of GCs are found in the spleen and the lymph nodes. Finally, lymphocytes infiltrating the SG are essentially MZ-like B cells. Note that BAFF-Tg mice develop the same pSS manifestations when T cells are removed [77], but in this instance, BAFF exacerbates Toll-like receptor activation of B cells. An alternative model for the development of SS apart from T cells has since been proposed [78].

### 3.10. CD5 in Its Implications in Autoimmunity

The CD5 is a transmembrane glycoprotein expressed in T lymphocytes and, at lower levels, in the subset of B cells known as B1 cell. The initial interest on CD5+ expressing B cells pointed on the role of these cells in autoimmune diseases, based on the ability of these cells to produce natural polyreactive antibodies, which recognize autoantigens with low affinity [79, 80]. The hypothesis in autoimmune diseases was that these natural antibodies with low affinity to autoantigens may improve this affinity and become in high affinity pathogenic autoantibodies. However, the B1 cells expressing CD5 have phenotypic features similar to transitional anergic murine B lymphocytes. In fact, these cells may produce IL-10 upon activation through the CD40 coreceptor [81, 82]. The regulatory potential of CD5 has been demonstrated by transfections of CD5 in Jok-1 B cell line [83]. In this experiment, the expression of CD5 induces IL-10 production through activating NFAT2 and STAT3. Thus CD5-expressing B cells may present contradictory roles in B lymphocytes function. An elegant study showing how CD5 expression is regulated in B lymphocytes and how it modulates the B cell response has been published. This study analyzed the molecular structure of the human CD5 gene, showing that two different promoters exist (E1A and E1B) [84]. The E1B Interestingly, the CD5-E1A was expressed on the membranes of both T and B lymphocytes, while the CD5-E1B was transcribed into a truncated CD5 isoform in B lymphocytes, not able to express on the membranes. CD5-E1B expression downregulates the level of membrane expression of the conventional CD5-C1A. As a consequence, high levels of CD5-E1B could reduce the inhibitory effects of CD5 on BCR mediated signaling and lead to increased antibody production. Thus, in B cells expressing high or normal levels of membrane CD5, the molecule acts to downregulate BCR mediated signaling. On the other hand, B cells expressing high levels of CD5-E1B, induced probably by external stimuli, would more likely to be activated. To support these results, in B lymphocytes from SLE patients, the levels of CD5-E1B are higher, indicating a more activating B cell [85]. These high levels of CD5-E1B traduces in reduced expression of membrane CD5. In this model, high levels of IL-6 on B cells from SLE patients abrogate the ability to induce the DNA methyl transferase (DNMT1) and then to methylate DNA, affecting the transcription of CD5-E1A, favoring the truncated form E1B. This altered signaling could promote the activation and expansion of autoreactive B cells in SLE patients. Interestingly, in mature B cells from SLE patients, a default in the regulation of *Rag* is present and leads to upregulation of this enzyme and the emergence of autoantibodies [86, 87]. CD5 and IL-6 contribute to this upregulation, indicating the roles of these molecules in SLE pathogenesis. 

In murine models CD5 is involved in anergy [88]. This hypothesis has been elegantly demonstrated breeding the HEL transgenic model for B cell anergy onto the CD5 null background. This experiment resulted in a spontaneous loss of B cell tolerance *in vivo*. The study showed high levels of anti-HEL IgM antibodies and enhanced proliferative responses *in vitro* with elevated intracellular calcium levels. 

### 3.11. CD22 in Its Implications in Autoimmunity

Another important B cell molecule which has an effect on autoimmunity development is the CD22. B cell responses are initiated by antigen binding to the BCR and are modified by a broad repertoire of activating and inhibitory transmembrane coreceptors expressed on the B cell surface [89, 90]. In this context, the multifunctional BCR co-receptor, CD22, is interesting since it plays a critical role in establishing and modulating the antigen receptor signaling thresholds for B cell activation [91]. CD22, as part of the BCR complex, can modulate the intensity, quality, and duration of homeostatic and BCR-induced signals in an inhibitory or stimulatory capacity through ligand-dependent and -independent mechanisms [92, 93]. Based on substantial mouse model data, it appears that the predominant effect of CD22 is inhibitory [94]. CD22 is a 135 kDa B lymphocyte restricted type-I transmembrane sialoglycoprotein of the immunoglobulin superfamily [95]. It appears intracellularly during the late pro-B cell stage of ontogeny but shifts to the plasma membrane with B cell maturation. CD22 is expressed at low levels on immature B cells and at higher levels on mature IgM+, IgD+ B cells. However, it is absent on differentiated plasma cells. It is strongly expressed in follicular, mantle, and marginal zone B cells but is weakly present in germinal B cells [96]. As previously mentioned, for the immune system to function effectively, it is essential to mount an appropriate humoral response against potential pathogens while avoiding autoimmunity and reactivity to self-antigens [97]. Understanding the function of CD22 may, therefore, suggest methods for modulating humoral immunity and aid in discovering treatments for autoimmunity [98]. 

To regulate B lymphocyte functions and migration, the interaction of CD22 with *α*2,6-linked sialic acid ligands is important. This binding is necessary for its negative regulatory functions [99]. Cell lines expressing CD22 without sialic acidbinding activity are hyperresponsive to BCR stimulation [99].

Recent studies in mouse models have suggested a role for defects and loss of functionality in CD22 in the pathogenesis of autoimmune disease, including SLE. B cells obtained from CD22-deficient mice have been shown to be hyperresponsive to receptor signaling and demonstrate increased Ca2+ fluxes on BCR ligation, which increased serum titers of IgG anti-dsDNA autoantibodies. These antibodies were of multiclonal origin, were somatically mutated, and had high affinity [100].

Epratuzumab is a novel humanized antihuman CD22 IgG1 monoclonal antibody that binds to the extracellular domain of CD22 and induces modest but significant intracellular phosphorylation. Epratuzumab reduces total blood B cells by about 35–40% and has preferential effects on naïve and transitional B cells [101, 102]. Epratuzumab treatment has been used with moderate clinical success in SLE and primary Sjögren's syndrome [103]. 

### 3.12. A New Concept in Autoimmunity: Regulatory B Cells

A functional B cell subset, called regulatory B cells, has recently emerged as an important factor for maintaining immune tolerance. This subtype restrains the excessive inflammatory response that occurs during the development of autoimmune diseases. The main regulatory B cell function is mediated by the IL-10 production that inhibits proinflammatory cytokines and supports regulatory T cell differentiation. The regulatory B cells were named in 2002 [104], after the demonstration that IL-10 producing B cells can suppress inflammatory responses in experimental autoimmune encephalomyelitis, collagen-induced arthritis, and autoimmune colitis [105, 106]. 

In the murine models, regulatory B cells have also been shown to directly inhibit T cell proliferation through cell-cell contact. This may even lead to anergy or apoptosis of T cells [107, 108] and the modulation of the inflammatory response. In this regard, CD40 engagement on B cells appears to be a requisite for the induction of functional B regulatory cells in mice. Stimulation of CD40 brings about the development of B cells with suppressive properties. Furthermore, signaling in the absence of CD40 makes B cells unable to regulate inflammatory response [105, 109].

The murine phenotypic nature of B regulatory cells is still a matter for debate. Two distinct IL-10 producing B cell subpopulations associated with regulatory functions have been identified. One has been recognized as transitional marginal zone precursor B cells expressing a high level of CD21, CD23, CD24, IgM, and CD1d, designed as transitional type 2 (T2)-like cells [110–112]. The second—described as CD1d^hi^, CD5+, and CD19^hi^ B cells—has been called “B10” cells since IL-10 is the main cytokine produced by these cells [81]. Recent studies have suggested that human B cells can also regulate inflammatory responses [113]. These cells have been studied primarily in autoimmune diseases, including SLE and multiple sclerosis, for which functional as well as numerical defects of these cells have been described [112, 114–116]. A recent publication on patients with SLE described a population of regulatory CD19+CD24++CD38++ B cells [112] as a phenotype reminiscent of preimmune B cells. This subset is able to secrete IL-10 and thus is able to suppress Th1 and Th2 functions after activation. These cells, though present in numbers similar to controls, lack regulatory capacity in SLE patients. 

In addition to the described results, another study on human regulatory B cells showed that regulation of T cell proliferation was defective in SLE patients but not in other autoimmune diseases [117]. This paper studied the regulation of T cell responses induced by B cells following CD40 cognate interaction. CD40-induced regulatory B cells partially inhibited T cell proliferation without any soluble factor. In contrast, modulation of Th1 differentiation resulted from CD80- and CD86-dependent interactions and IL-10 production. The suppressive effects were mediated by CD19++IgD+CD38++CD24++CD5++ and appeared to be indirect through the induction of regulatory T cells (CD4+CD25+FoxP3+). The mentioned defect of B cell regulatory effect was found only in SLE patients, indicating that the restoration of efficient B cell regulatory activity could be an innovative and alternative therapy in SLE. 

In another interesting paper from the same group, the effects of human B cells with a regulatory potential on dendritic cells have been studied. In an *in vitro* model of cocultures, human activated B cells (CD19+IgDlowCD38+CD24lowCD27−) showed a potential to restrain the development of monocytes into immature dendritic cells and their differentiation into mature dendritic cell, decreased the HLA-DR, CD80, and CD86 expression and the production of IL-12p70 required for antigen presentation and Th1 differentiation [118, 119]. Even more interesting, mature dendritic cells from patients with SLE displayed insensitivity to the regulation of IL-12 induced by B cells. Thus, inefficient B cell regulation may alter the balance between an effector inflammatory response and tolerance induction. 

Knowledge about these cells is increasing rapidly, but much remains to be understood regarding the biology of B regulatory cells in murine models and humans. The increasing knowledge may allow the development of targeted therapies in order to increase the B cell regulatory function in autoimmune diseases. 

### 3.13. B Cell Targeted Therapies in SLE

Several B cell molecules can be targeted to treat autoimmune diseases (Table 3). The most widely studied target for achieving B cell depletion in autoimmune disease is the CD20 antigen (human B cell-restricted differentiation antigen), a hydrophobic transmembrane protein with a molecular weight of approximately 35 kDa found on pre-B and mature B cells [120, 121] as well as in over 90% of the B cells in NHL [122]. Pilot studies of an anti-CD20 antibody (rituximab) in SLE were promising [123, 124]. A review of off-label use also suggested significant clinical and serological response [125]. However, two randomized trials showed no superiority of rituximab over standard therapy and did not reach primary or secondary end points [126, 127]. Despite these overall discouraging results, both studies have significant design shortcomings that limit their applicability. A study with another anti-CD20 antibody, ocrelizumab, was stopped prematurely due to an increase of serious infections. As mentioned above, CD22 inhibition with epratuzumab may be an alternative for B cell inhibition in SLE. A phase III study is now undergoing [128].

Another therapeutic approach is the inhibition of BAFF effects on B cell. This inhibition can be done by anti-BAFF or anti-BR3 monoclonal Abs, as well as BR3 or TACI decoy fusion proteins. Selective BAFF blockers prevent BAFF from interacting with its receptors, leaving APRIL available to interact with TACI and BCMA. Drugs in this class include anti-BAFF Ab (Belimumab or LymphoStat B) and a fusion protein consisting of human Ig Fc and of the extracellular BR3 domain (Briobacept, for BAFF-R-Ig). Nonselective BAFF blockers abolish the interactions of both BAFF and APRIL with all their receptors. To date, there is a single drug in this class which is human Ig Fc fused to the extracellular TACI domain (Atacicept, TACI-Ig). Differences in the distribution of the forms of BAFF could denote the potential of patients to respond or to resist to BAFF antagonist therapy. Treatment of B cells with TACI agonist Ab inhibits proliferation *in vitro, *and activation of a chimeric receptor containing TACI intracellular domain induces apoptosis. These results demonstrate also the critical requirement for TACI in regulating B cell homeostasis. The therapeutic effects of anti-BAFF therapy with Belimumab have been demonstrated in patients with SLE, based on two large randomized controlled trials, BLISS 52 and BLISS 76 [129].

## Figures and Tables

**Table 1:** Characteristics of primary B cell subsets and their progenitors.

Differentiation	Subset	Surface phenotype
Progenitor subsets (bone marrow)	Pro-B	B220loCD43+
		AA4.1+
	Pre-B	B220loCD43−, AA4.1+preBcR+
	Immature (23−)	B220lo, sIgM+, sIgD−, CD23−
	Immature (23+)	CD19+, B220+, sIgM+, sIgD−, CD23+
Transitional subsets (spleen)	T1	IgMhiCD23−, B220intAA4.1+
	T2	IgMhiCD23+, B220+AA4.1+
	T3	IgMloCD23+, B220+AA4.1+
Mature primary subsets	Follicular zone	IgMloCD23+, B220hiAA4.1−
	Marginal zone	CD19+IgMhiIgDlo CD23+ CD21+
	B1	CD43+ CD23− CD5+
T-independent responses	Early antibody-forming cells/short-lived plasma cells	B220loCD19+slg+iclghi
T-dependent responses	Early antibody-forming cells/short-lived plasma cells	B220loCD19+slg+iclghi
	Germinal center	B220+CD19+GL7+
	Long-lived plasma cells	B220loslg−iclg+
	Memory	B220+slg+IgD−
Natural antibodies	Peritoneal B1a and B1b	CD43+ CD23− CD5+

**Table 2:** Cell surface CD molecules that are preferentially expressed by B cells.

Name	Cellular reactivity	Structure
CD19	Pan-B cell, FDCs?	Ig superfamily
CD20	Mature B cells	MS4A family
CD21	Mature B cells, FDCs	Complement receptor family
CD22	Mature B cells	Ig superfamily
CD23	Activated B cells, FDCs, others	C-type lectin
CD24	Pan-B cell, granulocytes, epithelial cells	GPI anchored
CD40	B cells, epithelial cells, FDCs, others	TNF receptor
CD72	Pan-B cell	C-type lectin
CD79a,b	Surface Ig^+^ B cells	Ig superfamily

FDCs: follicular dendritic cells; Ig: immunoglobulin.

**Table 3:** Potential targets in B lymphocytes and the therapeutic molecule for the treatment of systemic lupus erythematosus.

Direct B lymphocyte targeting	
*CD-20 antigen *	
(i) Rituximab (chimeric monoclonal antibody): EXPLORER and LUNAR studies did not meet primary endpoints.	
(ii) Ocrelizumab (humanized monoclonal antibody): phase II prematurely stopped due to infections.	
(iii) Ofatumumab (human monoclonal antibody): no studies in SLE.	
(iv) Veltuzumab (humanized monoclonal antibody): no studies in SLE.	
(v) TRU-015 (engineered protein).	
*CD-22 antigen *	
(i) Epratuzumab (humanized monoclonal antibody anti-CD22): phase III study ongoing.	

Indirect B lymphocyte targeting	

*BAFF *	
(i) Belimumab (LimphoStat B: fully human monoclonal antibody anti-BAFF): FDA approved based on BLISS 52/BLISS 76 phase III studies.	
*BAFF receptors *	
(i) Anti-BR3.	
(ii) Atacicept (fusion IgG with the extracellular domain of TACI receptor): study in progress.	
(iii) Briobacept/BR3-Fc (fusion IgG with the extracellular domain of BAFF receptor—BR3).	

BAFF: B lymphocyte Activator Factor belonging to the TNF family; TACI: transmembrane activator and calcium modulator and cyclophylin ligand interactor.

## References

[1] Treml JF, Hao Y, Stadanlick JE, Cancro MP (2009). The BLyS family: toward a molecular understanding of B cell homeostasis. *Cell Biochemistry and Biophysics*.

[2] Bonilla FA, Oettgen HC (2010). Adaptive immunity. *Journal of Allergy and Clinical Immunology*.

[3] Fuxa M, Skok JA (2007). Transcriptional regulation in early B cell development. *Current Opinion in Immunology*.

[4] Lebien TW, Tedder TF (2008). B lymphocytes: how they develop and function. *Blood*.

[5] Wang YH, Liu YJ (2008). The IL-17 cytokine family and their role in allergic inflammation. *Current Opinion in Immunology*.

[6] Chaplin DD (2010). Overview of the immune response. *Journal of Allergy and Clinical Immunology*.

[7] Huston DP (1997). The biology of the immune system. *Journal of the American Medical Association*.

[8] Khan WN (2009). B cell receptor and BAFF receptor signaling regulation of B cell homeostasis. *Journal of Immunology*.

[9] Kurosaki T, Hikida M (2009). Tyrosine kinases and their substrates in B lymphocytes. *Immunological Reviews*.

[10] Le Pottier L, Devauchelle V, Pers J, Jamin C, Youinou P (2007). The mosaic of B-cell subsets (with special emphasis on primary Sjögren’s syndrome). *Autoimmunity Reviews*.

[11] Palanichamy A, Barnard J, Zheng B (2009). Novel human transitional B cell populations revealed by B cell depletion therapy. *Journal of Immunology*.

[12] Schiemann B, Gommerman JL, Vora K (2001). An essential role for BAFF in the normal development of B cells through a BCMA-independent pathway. *Science*.

[13] Chung JB, Sater RA, Fields ML, Erikson J, Monroe JG (2002). CD23 defines two distinct subsets of immature B cells which differ in their responses to T cell help signals. *International Immunology*.

[14] Saito T, Chiba S, Ichikawa M (2003). Notch2 is preferentially expressed in mature B cells and indispensable for marginal zone B lineage development. *Immunity*.

[15] Willenbrock K, Jungnickel B, Hansmann M, Küppers R (2005). Human splenic marginal zone B cells lack expression of activation-induced cytidine deaminase. *European Journal of Immunology*.

[16] Reif K, Ekland EH, Ohl L (2002). Balanced responsiveness to chemoattractants from adjacent zones determines B-cell position. *Nature*.

[17] Cinamon G, Zachariah MA, Lam OM, Foss FW, Cyster JG (2008). Follicular shuttling of marginal zone B cells facilitates antigen transport. *Nature Immunology*.

[18] Allen CDC, Cyster JG (2008). Follicular dendritic cell networks of primary follicles and germinal centers: phenotype and function. *Seminars in Immunology*.

[19] Pillai S, Cariappa A (2009). The follicular versus marginal zone B lymphocyte cell fate decision. *Nature Reviews Immunology*.

[20] Bende RJ, Van Maldegem F, Van Noesel CJM (2009). Chronic inflammatory disease, lymphoid tissue neogenesis and extranodal marginal zone B-cell lymphomas. *Haematologica*.

[21] Hart GT, Wang X, Hogquist KA, Jameson SC (2011). Krüppel-like factor 2 (KLF2) regulates B-cell reactivity, subset differentiation, and trafficking molecule expression. *Proceedings of the National Academy of Sciences of the United States of America*.

[22] Hoek KL, Gordy LE, Collins PL (2010). Follicular B cell trafficking within the spleen actively restricts humoral immune responses. *Immunity*.

[23] Goetzl EJ, Wang W, McGiffert C, Huang M, Gräler MH (2004). Sphingosine 1-phosphate and its G protein-coupled receptors constitute a multifunctional immunoregulatory system. *Journal of Cellular Biochemistry*.

[24] Wang JH, Wu Q, Yang P (2011). Type i interferon-dependent CD86^high^ marginal zone precursor b cells are potent T cell costimulators in mice. *Arthritis and Rheumatism*.

[25] Shi G, Harrison K, Wilson GL, Moratz C, Kehrl JH (2002). RGS13 regulates germinal center B lymphocytes responsiveness to CXC chemokine ligand (CXCL)12 and CXCL13. *Journal of Immunology*.

[26] Pascual V, Liu Y, Magalski A, De Bouteiller O, Banchereau J, Capra JD (1994). Analysis of somatic mutation in five B cell subsets of human tonsil. *Journal of Experimental Medicine*.

[27] Odendahl M, Jacobi A, Hansen A (2000). Disturbed peripheral B lymphocyte homeostasis in systemic lupus erythematosus. *Journal of Immunology*.

[28] Lee J, Kuchen S, Fischer R, Chang S, Lipsky PE (2009). Identification and characterization of a human CD5+ pre-naive B cell population. *Journal of Immunology*.

[29] Sims GP, Ettinger R, Shirota Y, Yarboro CH, Illei GG, Lipsky PE (2005). Identification and characterization of circulating human transitional B cells. *Blood*.

[30] Bohnhorst JO, Bjørgan MB, Thoen JE, Natvig JB, Thompson KM (2001). Bm1-Bm5 classification of peripheral blood B cells reveals circulating germinal center founder cells in healthy individuals and disturbance in the B cell subpopulations in patients with primary Sjögren’s syndrome. *Journal of Immunology*.

[31] Harada Y, Kawano MM, Huang N (1996). Identification of early plasma cells in peripheral blood and their clinical significance. *British Journal of Haematology*.

[32] Jacobi AM, Odendahl M, Reiter K (2003). Correlation between circulating CD27^high^ plasma cells and disease activity in patients with systemic lupus erythematosus. *Arthritis and Rheumatism*.

[33] Parrish YK, Baez I, Milford T (2009). IL-7 dependence in human B lymphopoiesis increases during progression of ontogeny from cord blood to bone marrow. *Journal of Immunology*.

[34] Giliani S, Mori L, De Saint Basile G (2005). Interleukin-7 receptor *α* (IL-7R*α*) deficiency: cellular and molecular bases. Analysis of clinical, immunological, and molecular features in 16 novel patients. *Immunological Reviews*.

[35] Yoshimura A, Naka T, Kubo M (2007). SOCS proteins, cytokine signalling and immune regulation. *Nature Reviews Immunology*.

[36] Youinou P, Taher TE, Pers J, Mageed RA, Renaudineau Y (2009). B lymphocyte cytokines and rheumatic autoimmune disease. *Arthritis and Rheumatism*.

[37] Mitsias DI, Tzioufas AG, Veiopoulou C (2002). The Th1/Th2 cytokine balance changes with the progress of the immunopathological lesion of Sjogren’s syndrome. *Clinical and Experimental Immunology*.

[38] Szodoray P, Alex P, Brun JG, Centola M, Jonsson R (2004). Circulating cytokines in primary Sjögren’s syndrome determined by a multiplex cytokine array system. *Scandinavian Journal of Immunology*.

[39] Fuxa M, Busslinger M (2007). Reporter gene insertions reveal a strictly B lymphoid-specific expression pattern of Pax5 in support of its B cell identity function. *Journal of Immunology*.

[40] Yurasov S, Wardemann H, Hammersen J (2005). Defective B cell tolerance checkpoints in systemic lupus erythematosus. *Journal of Experimental Medicine*.

[41] Meyersa G, Nga YS, Bannocka JM (2011). Activation-induced cytidine deaminase (AID) is required for B-cell tolerance in humans. *Proceedings of the National Academy of Sciences of the United States of America*.

[42] Luning Prak ET, Monestier M, Eisenberg RA (2011). B cell receptor editing in tolerance and autoimmunity. *Annals of the New York Academy of Sciences*.

[43] Bergman Y, Cedar H (2004). A stepwise epigenetic process controls immunoglobulin allelic exclusion. *Nature Reviews Immunology*.

[44] Lindsley RC, Thomas M, Srivastava B, Allman D (2007). Generation of peripheral B cells occurs via two spatially and temporally distinct pathways. *Blood*.

[45] Bräuninger A, Goossens T, Rajewsky K, Küppers R (2001). Regulation of immunoglobulin light chain gene rearrangements during early B cell development in the human. *European Journal of Immunology*.

[46] Klonowski KD, Primiano LL, Monestier M (1999). Atypical V(H)-D-J(H) rearrangements in newborn autoimmune MRL mice. *Journal of Immunology*.

[47] Klonowski KD, Monestier M (2000). Heavy chain revision in MRL mice: a potential mechanism for the development of autoreactive B cell precursors. *Journal of Immunology*.

[48] Derudder E, Cadera EJ, Vahl JC (2009). Development of immunoglobulin *λ*-chain-positive B cells, but not editing of immunoglobulin *κ*-chain, depends on NF-*κ*B signals. *Nature Immunology*.

[49] Benschop RJ, Melamed D, Nemazee D, Cambier JC (1999). Distinct signal thresholds for the unique antigen receptor-linked gene expression programs in mature and immature B cells. *Journal of Experimental Medicine*.

[50] Verkoczy L, Duong B, Skog P (2007). Basal B cell receptor-directed phosphatidylinositol 3-kinase signaling turns off RAGs and promotes B cell-positive selection. *Journal of Immunology*.

[51] Shlomchik MJ, Marshak-Rothstein A, Wolfowicz CB (1987). The role of clonal selection and somatic mutation in autoimmunity. *Nature*.

[52] Litzenburger T, Bluthmann H, Morales P (2000). Development of myelin oligodendrocyte glycoprotein autoreactive transgenic B lymphocytes: receptor editing in vivo after encounter of a self-antigen distinct from myelin oligodendrocyte glycoprotein. *Journal of Immunology*.

[53] Litzenburger T, Fässler R, Bauer J (1998). B lymphocytes producing demyelinating autoantibodies: development and function in gene-targeted transgenic mice. *Journal of Experimental Medicine*.

[54] Menard L, Saadoun D, Isnardi I (2011). The PTPN22 allele encoding an R620W variant interferes with the removal of developing autoreactive B cells in humans. *Journal of Clinical Investigation*.

[55] Inaoki M, Sato S, Weintraub BC, Goodnow CC, Tedder TF (1997). CD19-regulated signaling thresholds control peripheral tolerance and autoantibody production in B lymphocytes. *Journal of Experimental Medicine*.

[56] Guerrier T, Youinou P, Pers JO, Jamin C (2012). TLR9 drives the development of transitional B cells towards the marginal zone pathway and promotes autoimmunity. *Journal of Autoimmunity*.

[57] Lyons JA, San M, Happ MP, Cross AH (1999). B cells are critical to induction of experimental allergic encephalomyelitis by protein but not by a short encephalitogenic peptide. *European Journal of Immunology*.

[58] Bettelli E, Baeten D, Jäger A, Sobel RA, Kuchroo VK (2006). Myelin oligodendrocyte glycoprotein-specific T and B cells cooperate to induce a Devic-like disease in mice. *Journal of Clinical Investigation*.

[59] Krishnamoorthy G, Lassmann H, Wekerle H, Holz A (2006). Spontaneous opticospinal encephalomyelitis in a double-transgenic mouse model of autoimmune T cell/B cell cooperation. *Journal of Clinical Investigation*.

[60] Monson NL, Cravens P, Hussain R (2011). Rituximab therapy reduces organ-specific T cell responses and ameliorates experimental autoimmune encephalomyelitis. *PLoS ONE*.

[61] Chan OT, Hannum LG, Haberman AM, Madaio MP, Shlomchik MJ (1999). A novel mouse with B cells but lacking serum antibody reveals an antibody-independent role for B cells in murine lupus. *Journal of Experimental Medicine*.

[62] Wong FS, Wen L, Tang M (2004). Investigation of the role of B-cells in type 1 diabetes in the NOD mouse. *Diabetes*.

[63] Tchernev G, Orfanos CE (2006). Antigen mimicry, epitope spreading and the pathogenesis of pemphigus. *Tissue Antigens*.

[64] Sokolove J, Bromberg R, Deane KD (2012). Autoantibody epitope spreading in the pre-clinical phase predicts progression to rheumatoid arthritis. *PLoS One*.

[65] McRae BL, Vanderlugt CL, Dal Canto MC, Miller SD (1995). Functional evidence for epitope spreading in the relapsing pathology of experimental autoimmune encephalomyelitis. *Journal of Experimental Medicine*.

[66] Tisch R, Yang X-D, Singer SM, Liblau RS, Fugger L, McDevitt HO (1993). Immune response to glutamic acid decarboxylase correlates with insulitis in non-obese diabetic mice. *Nature*.

[67] Ziegler AG, Hummel M, Schenker M, Bonifacio E (1999). Autoantibody appearance and risk for development of childhood diabetes in offspring of parents with type 1 diabetes: the 2-year analysis of the German BABYDIAB Study. *Diabetes*.

[68] van der Woude D, Rantapää-Dahlqvist S, Ioan-Facsinay A (2010). Epitope spreading of the anti-citrullinated protein antibody response occurs before disease onset and is associated with the disease course of early arthritis. *Annals of the Rheumatic Diseases*.

[69] Arbuckle MR, McClain MT, Rubertone MV (2003). Development of autoantibodies before the clinical onset of systemic lupus erythematosus. *New England Journal of Medicine*.

[70] Schneider P, Mackay F, Steiner V (1999). BAFF, a novel ligand of the tumor necrosis factor family, stimulates B cell growth. *Journal of Experimental Medicine*.

[71] Mackay F, Browning JL (2002). BAFF: a fundamental survival factor for B cells. *Nature Reviews Immunology*.

[72] Mackay F, Woodcock SA, Lawton P (1999). Mice transgenic for BAFF develop lymphocytic disorders along with autoimmune manifestations. *Journal of Experimental Medicine*.

[73] Brink R (2006). Regulation of B cell self-tolerance by BAFF. *Seminars in Immunology*.

[74] Schiemann B, Gommerman JL, Vora K (2001). An essential role for BAFF in the normal development of B cells through a BCMA-independent pathway. *Science*.

[75] Lesley R, Xu Y, Kalled SL (2004). Reduced competitiveness of autoantigen-engaged B cells due to increased dependence on BAFF. *Immunity*.

[76] Thien M, Phan TG, Gardam S (2004). Excess BAFF rescues self-reactive B cells from peripheral deletion and allows them to enter forbidden follicular and marginal zone niches. *Immunity*.

[77] Groom JR, Fletcher CA, Walters SN (2007). BAFF and MyD88 signals promote a lupuslike disease independent of T cells. *Journal of Experimental Medicine*.

[78] Mackay F, Groom JR, Tangye SG (2007). An important role for B-cell activation factor and B cells in the pathogenesis of Sjögren’s syndrome. *Current Opinion in Rheumatology*.

[79] Youinou P, Jamin C, Lydyard PM (1999). CD5 expression in human B-cell populations. *Immunology Today*.

[80] Berland R, Wortis HH (2002). Origins and functions of B-1 cells with notes on the role of CD5. *Annual Review of Immunology*.

[81] Yanaba K, Bouaziz JD, Haas KM, Poe JC, Fujimoto M, Tedder TF (2008). A regulatory B cell subset with a unique CD1d^hi^CD5^+^ phenotype controls T cell-dependent inflammatory responses. *Immunity*.

[82] Mauri C, Gray D, Mushtaq N, Londei M (2003). Prevention of arthritis by interleukin 10-producing B cells. *Journal of Experimental Medicine*.

[83] Garaud S, Morva A, Lemoine S (2011). CD5 promotes IL-10 production in chronic lymphocytic leukemia B cells through STAT3 and NFAT2 activation. *Journal of Immunology*.

[84] Renaudineau Y, Hillion S, Saraux A, Mageed RA, Youinou P (2005). An alternative exon 1 of the CD5 gene regulates CD5 expression in human B lymphocytes. *Blood*.

[85] Garaud S, Le Dantec C, Jousse-Joulin S (2009). IL-6 Modulates CD5 expression in B cells from patients with lupus by regulating DNA methylation. *Journal of Immunology*.

[86] Hillion S, Saraux A, Youinou P, Jamin C (2005). Expression of RAGs in peripheral B cells outside Germinal Centers is associated with the expression of CD5. *Journal of Immunology*.

[87] Hillion S, Dueymes M, Youinou P, Jamin C (2007). IL-6 contributes to the expression of RAGs in human mature B cells. *Journal of Immunology*.

[88] Hippen KL, Tze LE, Behrens TW (2000). CD5 maintains tolerance in anergic B cells. *Journal of Experimental Medicine*.

[89] Poe JC, Hasegawa M, Tedder TF (2001). CD19, CD21, and CD22: multifaceted response regulators of B lymphocyte signal transduction. *International Reviews of Immunology*.

[90] Pritchard NR, Smith KGC (2003). B cell inhibitory receptors and autoimmunity. *Immunology*.

[91] Cyster JG, Goodnow CC (1997). Tuning antigen receptor signaling by CD22: integrating cues from antigens and the microenvironment. *Immunity*.

[92] Tedder TF, Tuscano J, Sato S, Kehrl JH (1997). CD22, A B lymphocyte-specific adhesion molecule that regulates antigen receptor signaling. *Annual Review of Immunology*.

[93] Sato S, Miller AS, Inaoki M (1996). CD22 is both a positive and negative regulator of B lymphocyte antigen receptor signal transduction: altered signaling in CD22-deficient mice. *Immunity*.

[94] Nitschke L (2005). The role of CD22 and other inhibitory co-receptors in B-cell activation. *Current Opinion in Immunology*.

[95] Engel P, Nojima Y, Rothstein D (1993). The same epitope on CD22 of B lymphocytes mediates the adhesion of erythrocytes, T and B lymphocytes, neutrophils, and monocytes. *Journal of Immunology*.

[96] Tedder TF, Poe JC, Haas KM (2005). CD22: a multifunctional receptor that regulates B lymphocyte survival and signal transduction. *Advances in Immunology*.

[97] Grimaldi CM, Hicks R, Diamond B (2005). B cell selection and susceptibility to autoimmunity. *Journal of Immunology*.

[98] Walker JA, Smith KGC (2008). CD22: an inhibitory enigma. *Immunology*.

[99] Jin L, McLean PA, Neel BG, Wortis HH (2002). Sialic acid binding domains of CD22 are required for negative regulation of B cell receptor signaling. *Journal of Experimental Medicine*.

[100] O’Keefe TL, Williams GT, Davies SL, Neuberger MS (1996). Hyperresponsive B cells in CD22-deficient mice. *Science*.

[101] Dörner T, Kaufmann J, Wegener WA, Teoh N, Goldenberg DM, Burmester GR (2006). Initial clinical trial of epratuzumab (humanized anti-CD22 antibody) for immunotherapy of systemic lupus erythematosus. *Arthritis Research and Therapy*.

[102] Jacobi AM, Goldenberg DM, Hiepe F, Radbruch A, Burmester GR, Dörner T (2008). Differential effects of epratuzumab on peripheral blood B cells of patients with systemic lupus erythematosus versus normal controls. *Annals of the Rheumatic Diseases*.

[103] Steinfeld SD, Tant L, Burmester GR (2006). Epratuzumab (humanised anti-CD22 antibody) in primary Sjögren’s syndrome: an open-label phase I/II study. *Arthritis Research and Therapy*.

[104] Mizoguchi A, Mizoguchi E, Takedatsu H, Blumberg RS, Bhan AK (2002). Chronic intestinal inflammatory condition generates IL-10-producing regulatory B cell subset characterized by CD1d upregulation. *Immunity*.

[105] Fillatreau S, Sweenie CH, McGeachy MJ, Gray D, Anderton SM (2002). B cells regulate autoimmunity by provision of IL-10. *Nature Immunology*.

[106] Mauri C, Gray D, Mushtaq N, Londei M (2003). Prevention of arthritis by interleukin 10-producing B cells. *Journal of Experimental Medicine*.

[107] Wei B, Velazquez P, Turovskaya O (2005). Mesenteric B cells centrally inhibit CD4^+^ T cell colitis through interaction with regulatory T cell subsets. *Proceedings of the National Academy of Sciences of the United States of America*.

[108] Tretter T, Venigalla RKC, Eckstein V (2008). Induction of CD4^+^ T-cell anergy and apoptosis by activated human B cells. *Blood*.

[109] Mizoguchi A, Mizoguchi E, Smith RN, Preffer FI, Bhan AK (1997). Suppressive role of B cells in chronic colitis of T cell receptor *α* mutant mice. *Journal of Experimental Medicine*.

[110] Evans JG, Chavez-Rueda KA, Eddaoudi A (2007). Novel suppressive function of transitional 2 B cells in experimental arthritis. *Journal of Immunology*.

[111] Blair PA, Chavez-Rueda KA, Evans JG (2009). Selective targeting of B cells with agonistic anti-CD40 is an efficacious strategy for the generation of induced regulatory T2-like B cells and for the suppression of lupus in MRL/lpr mice. *Journal of Immunology*.

[112] Blair PA, Noreña LY, Flores-Borja F (2010). CD19^+^CD24^hi^CD38^hi^ B cells exhibit regulatory capacity in healthy individuals but are functionally impaired in systemic lupus erythematosus patients. *Immunity*.

[113] Jamin C, Morva A, Lemoine S, Daridon C, De Mendoza AR, Youinou P (2008). Regulatory B lymphocytes in humans: a potential role in autoimmunity. *Arthritis and Rheumatism*.

[114] Duddy M, Niino M, Adatia F (2007). Distinct effector cytokine profiles of memory and naive human B cell subsets and implication in multiple sclerosis. *Journal of Immunology*.

[115] Correale J, Farez M, Razzitte G (2008). Helminth infections associated with multiple sclerosis induce regulatory B cells. *Annals of Neurology*.

[116] Mauri C (2010). Regulation of immunity and autoimmunity by B cells. *Current Opinion in Immunology*.

[117] Lemoine S, Morva A, Youinou P, Jamin C (2011). Human T cells induce their own regulation through activation of B cells. *Journal of Autoimmunity*.

[118] Morva A, Lemoine S, Achour A, Pers J, Youinou P, Jamin C (2012). Maturation and function of human dendritic cells are regulated by B lymphocytes. *Blood*.

[119] Lemoine S, Morva A, Youinou P, Jamin C (2009). Regulatory B cells in autoimmune diseases: how do they work. *Annals of the New York Academy of Sciences*.

[120] Einfeld DA, Brown JP, Valentine MA, Clark EA, Ledbetter JA (1988). Molecular cloning of the human B cell CD20 receptor predicts a hydrophobic protein with multiple transmembrane domains. *EMBO Journal*.

[121] Valentine MA, Meier KE, Rossie S, Clark EA (1989). Phosphorylation of the CD20 phosphoprotein in resting B lymphocytes. Regulation by protein kinase C. *Journal of Biological Chemistry*.

[122] Anderson KC, Bates MP, Slaughenhoupt BL (1984). Expression of human B cell-associated antigens on leukemias and lymphomas: a model of human B cell differentiation. *Blood*.

[123] Anolik JH, Barnard J, Cappione A (2004). Rituximab improves peripheral B cell abnormalities in human systemic lupus erythematosus. *Arthritis and Rheumatism*.

[124] Leandro MJ, Edwards JC, Cambridge G, Ehrenstein MR, Isenberg DA (2002). An open study of B lymphocyte depletion in systemic lupus erythematosus. *Arthritis and Rheumatism*.

[125] Ramos-Casals M, Soto MJ, Cuadrado MJ, Khamashta MA (2009). Rituximab in systemic lupus erythematosus A systematic review of off-label use in 188 cases. *Lupus*.

[126] Merrill J, Buyon J, Furie R (2011). Assessment of flares in lupus patients enrolled in a phase II/III study of rituximab (EXPLORER). *Lupus*.

[127] Rovin BH, Furie R, Latinis K (2012). Efficacy and safety of rituximab in patients with active proliferative lupus nephritis: the Lupus Nephritis Assessment with Rituximab study. *Arthritis & Rheumatism*.

[128] Daridon C, Blassfeld D, Reiter K (2010). Epratuzumab targeting of CD22 affects adhesion molecule expression and migration of B-cells in systemic lupus erythematosus. *Arthritis Research and Therapy*.

[129] Navarra SV, Guzmán RM, Gallacher AE (2011). Efficacy and safety of belimumab in patients with active systemic lupus erythematosus: a randomised, placebo-controlled, phase 3 trial. *The Lancet*.

